# Effective treatment of *Clostridioides difficile* infection improves survival and affects graft-versus-host disease: a multicenter study by the Polish Adult Leukemia Group

**DOI:** 10.1038/s41598-024-56336-3

**Published:** 2024-03-11

**Authors:** Agnieszka Piekarska, Alicja Sadowska-Klasa, Patrycja Mensah-Glanowska, Małgorzata Sobczyk-Kruszelnicka, Joanna Drozd-Sokołowska, Anna Waszczuk-Gajda, Joanna Kujawska, Mateusz Wilk, Agnieszka Tomaszewska, Jan M. Zaucha, Sebastian Giebel, Lidia Gil

**Affiliations:** 1 Department of Hematology and Transplantology, Medical University of Gdańsk and University Clinical Center, ul. Smoluchowskiego 17, 80-214 Gdańsk, Poland; 2 Department of Hematology, Jagiellonian University Collegium Medicum, University Hospital in Cracow, Cracow, Poland; 3 Department of Bone Marrow Transplantation and Onco-Hematology, Maria Sklodowska-Curie Institute - Oncology Center, Gliwice Branch, Gliwice, Poland; 4 Department of Hematology, Transplantation and Internal Medicine, Medical University of Warsaw, Warsaw, Poland; 5 Department of Hematology and Bone Marrow Transplantation, Poznan University of Medical Sciences, Poznań, Poland; 6 Department of Hematology, University Hospital in Cracow, Cracow, Poland

**Keywords:** Allogeneic hematopoietic cell transplantation, *Clostridioides difficile* infection, Graft-versus-host disease, Metronidazole, Vancomycin, Stem cells, Diseases, Medical research

## Abstract

*Clostridioides difficile* infection (CDI) is the most common cause of infectious diarrhea after allogeneic hematopoietic cell transplantation (allo-HCT). The impact of CDI and its treatment on allo-HCT outcomes and graft-versus-host disease (GVHD), including gastrointestinal GVHD (GI-GVHD) is not well established. This multicenter study assessed real-life data on the first-line treatment of CDI and its impact on allo-HCT outcomes. Retrospective and prospective data of patients with CDI after allo-HCT were assessed. We noted statistically significant increase in the incidence of acute GVHD and acute GI-GVHD after CDI (*P* = 0.005 and *P* = 0.016, respectively). The first-line treatment for CDI included metronidazole in 34 patients, vancomycin in 64, and combination therapy in 10. Treatment failure was more common with metronidazole than vancomycin (38.2% vs. 6.2%; *P* < 0.001). The need to administer second-line treatment was associated with the occurrence or exacerbation of GVHD (*P* < 0.05) and GI-GVHD (*P* < 0.001) and reduced overall survival (*P* < 0.05). In the multivariate analysis, the risk of death was associated with acute GVHD presence before CDI (hazard ratio [HR], 3.19; *P* = 0.009) and the need to switch to second-line treatment (HR, 4.83; *P* < 0.001). The efficacy of the initial CDI treatment affects survival and occurrence of immune-mediated GI-GVHD after allo-HCT. Therefore, agents with higher efficacy than metronidazole (vancomycin or fidaxomicin) should be administered as the first-line treatment.

## Introduction

*Clostridioides difficile* infection (CDI) is caused by this bacterium overgrowth in intestinal microbiota disturbed by antibiotic use. It is one of the most common causes of infectious diarrhea in patients after allogeneic hematopoietic cell transplantation (allo-HCT), with the reported incidence ranging from 9 to 27%^1–8^. Patients after allo-HCT are more susceptible to CDI than the general population owing to the disruption of the mucosa and bacterial microbiota caused by the conditioning regimens, exposure to prophylactic and therapeutic use of broad-spectrum antibiotics, immunosuppression, and prolonged hospitalizations^9,10^.

According to previous guidelines published by the European Society of Clinical Microbiology, Infectious Diseases Society of America (IDSA), and Society for Healthcare Epidemiology of America (SHEA) for the general population, the choice of a therapeutic agent should be guided by disease severity and a recurrence history^11–13^. The risk factors for severe CDI include underlying disease and immunodeficiency, which places allo-HCT recipients in the high-risk group where vancomycin or fidaxomicin should be given in the first line, even in non-severe CDI^11,14^. This was reiterated in the 2021 update of IDSA/SHEA recommendations and the first practice guidelines for CDI management in HCT recipients of the American Society for Transplantation and Cellular Therapy (ASCTC) published in 2022^15,16^.

Despite these recommendations, a recent survey by the Infectious Diseases Working Party of the European Society for Blood and Marrow Transplantation (IDWP-EBMT) revealed that metronidazole was used in numerous transplant centers, even in severe or recurrent CDI^17^. According to pharmacokinetic data, oral vancomycin achieves the higher activity than metronidazole in the colon due its poor absorption^18,19^. Additionally, antibiotics unselectively targeting *Clostridiales* strains beneficial for T-regulatory cell formation may enhance pro-inflammatory processes in the guts leading to the development of graft-versus-host disease (GVHD) or to disease exacerbations if GVHD was present before CDI^20^.

The occurrence of CDI can be related to GVHD, especially gastrointestinal GVHD (GI-GVHD). The presence of GVHD and its treatment cause immunity defects and increase the occurrence of infection, leading to antibiotic exposure. On the other hand, ineffective CDI treatment may lead to persistence of inflammation and damage exposing human leukocyte antigens to immune cells caused by bacterial toxins, which, in turn, might influence alloreactivity^21,22^.

The impact of CDI and its treatment on allo-HCT outcomes is not well established. Associations between CDI and GI-GVHD are also unclear. Therefore, the Polish Adult Leukemia Group (PALG) performed a study with the aim to:: (1) assess the efficacy of CDI treatment in immunocompromised patients after allo-HCT; and (2) investigate the impact of CDI and the type of CDI treatment on allo-HCT outcomes and the occurrence of GI-GVHD.

## Patients and methods

### Study design

All allo-HCT recipients with documented CDI were eligible for the study. First, data for CDI patients subjected to allo-HCT between 2012 and 2016 were collected retrospectively from PALG transplant centers. In 2017, a prospective protocol was initiated in the transplant centers with the administration of oral vancomycin as the first-line treatment of nonsevere and severe manifestations of CDI and a combination therapy with oral or rectal vancomycin and intravenous (IV) metronidazole in fulminant CDI. In the case of treatment failure, fidaxomicin was recommended. This was a noninterventional study, and the final choice of a therapeutic agent was at the discretion of the treating physician. In December 2021, we collected a second round of data on patients diagnosed with CDI between 2017 and 2021. Patients with *C. difficile* colonization without symptoms of CDI were excluded.

The primary endpoints were a CDI remission rate after the first-line treatment with metronidazole, vancomycin, fidaxomicin, or combination therapy and correlations between CDI treatment and GI-GVHD. The secondary endpoints were overall survival (OS) and non-relapse mortality (NRM) in patients with CDI as well as changes in clinical practice after introducing the noninterventional PALG protocol for CDI treatment.

### Definitions

The diagnosis of CDI was based on symptoms (new-onset diarrhea or acute worsening of chronic diarrhea) and a positive toxin test or positive glutamate dehydrogenase test confirmed by the nucleic acid amplification test in feces or positive culture^23^.

Clinical cure was defined as resolution of diarrhea and no need for CDI treatment after completion of the therapy. The cure rate analysis did not include patients who died for another reason than CDI before completing the CDI treatment. These cases were reported as deaths with active CDI.

The criterion for engraftment was the first day of the 3 days with an absolute neutrophil count of higher than 0.5 × 10^9^/L.

Acute GVHD (aGVHD) was diagnosed according to Mount Sinai Acute GvHD International Consortium criteria^24^. Gastrointestinal (GI) involvement was verified with histopathological examination. Chronic GVHD (cGVHD) grading were based on 2014 National Institutes of Health Consensus Criteria^25^.

### Ethics approval

The study was conducted in accordance with the latest version of the Declaration of Helsinki. The local Human Research Ethics Committee of the Medical University of Gdansk approved the publication of the study because patient-identifying data were omitted to protect anonymity, and the microbiological samples were collected as routine tests with a prior informed consent of patients, available in the patients’ medical records (Approval Number, NKBBN/16/2021).

### Statistical analysis

Categorical variables were expressed as absolute numbers with percentages, and the differences between groups were compared using the Pearson’s χ^2^ test. Continuous variables were expressed as median values with ranges. The relationship between continuous and categorical variables was assessed using the nonparametric Mann–Whitney test. Survival analysis was performed using the Kaplan–Meier method. Overall survival was calculated from the date of CDI diagnosis until death from any cause. The study population was stratified according to principal clinical and demographic characteristics, and the mean values were compared using the log-rank test. A multivariate Cox regression analysis was applied to identify independent predictive factors for the risk of death analysis. A *P* value of less than 0.05 was considered significant. All analyses were performed using STATISTICA version 12 (StatSoft, Inc., Tulsa, Oklahoma, United States).

## Results

### Characteristics of the study population

The detailed characteristics of the 109 patients who underwent allo-HCT between 2012 and 2021, including 68 patients with CDI diagnosed after 2016, are presented in Table 1.

**Table 1:** Characteristics of the study group.

Parameter		All patients	Patients diagnosed 2012–2016	Patients diagnosed 2017–2021
Demographic characteristics				
Group size, n (%)		109 (100)	41 (37.6)	68 (62.3)
Sex distribution: female/male n (%)		54 (49.5)/55 (50.5)	19 (46.3)/22 (53.7)	35 (51.5)/33 (48.5)
Age at allo-HCT, y, median (range)		49 (18–72)	45 (20–65)	51 (18–72)
Diagnosis, n (%)				
AML		51 (46.7)	20 (48.7)	31 (45.6)
ALL		26 (23.9)	9 (22.0)	17 (25.0)
MPN/MDS		20 (18.3)	9 (22.0)	11 (16.2)
MM/HL/NHL/CLL		12 (11.0)	3 (7.3)	9 (13.2)
HCT-CI (n = 106), n (%)				
0		45 (42.4)	21 (51.2)	24 (36.9)
1–2		34 (32.1)	16 (39.0)	18 (27.7)
3–5		27 (25.5)	4 (9.8)	23 (35.4)
Transplant characteristics				
Donor type, n (%)	Matched sibling donor	32 (29.4)	16 (39.0)	16 (23.5)
	Unrelated donor (matched; mismatched)	65 (59.6)	22 (53.7)	43 (63.2)
	Haploidentical	12 (11.0)	3 (7.3)	9 (13.2)
Graft source: PB/BM, n (%)		106 (97.2)/3 (2.8)	40 (97.6)/1 (2.4)	66 (97.0)/2 (3.0)
Conditioning regimen: MAC or RTC/RIC, n (%)		72 (66)/37 (34)	32 (78)/9 (22)	40 (58.8)/28 (41.2)
Day of neutrophil engraftment ANC > 0.5 G/l (if achieved), median (range)		17 (6–43)	19 (10–43)	17 (6–43)
Engraftment not achieved, n (%)		4 (3.7)	1 (2.4)	3 (4.4)

ALL, Acute lymphoblastic leukemia; AML, Acute myeloid leukemia; ANC, Absolute neutrophil count; BM, Bone marrow; CDI, *Clostridioides difficile* infection; CLL, Chronic lymphocytic leukemia; HCT, Hematopoietic cell transplantation; HCT-CI, HCT comorbidity index; HL, Hodgkin lymphoma; MAC, Myeloablative conditioning; MDS, Myelodysplastic neoplasm; MM, Multiple myeloma; MPN, Myeloproliferative neoplasm; NHL, Non-Hodgkin lymphoma; PB, Peripheral blood; RIC, Reduced-intensity conditioning; RTC, Reduced-toxicity conditioning.

### Diagnosis of *Clostridioides difficile* infection and outcomes of first-line and second-line treatment

Infections with CDI were diagnosed a median of 11 days after allo-HCT (range, − 13 to 740 days). Twenty-four cases (18.3%) were diagnosed in the peritransplant period (just before or during the conditioning regimen up to day 0 of graft infusion). In most of the remaining cases (n = 64; 58.7%), CDI developed before day 100 after allo-HCT. The diagnosis of CDI was established based on recommended clinical criteria and laboratory confirmation of the presence of toxin A (n = 6; 5.5%), toxin B (n = 12; 11%), both toxins A and B (n = 58; 53.2%), or glutamate dehydrogenase confirmed by the positive nucleic acid amplification test or positive culture (n = 32; 29.3%).

**Figure 1:**
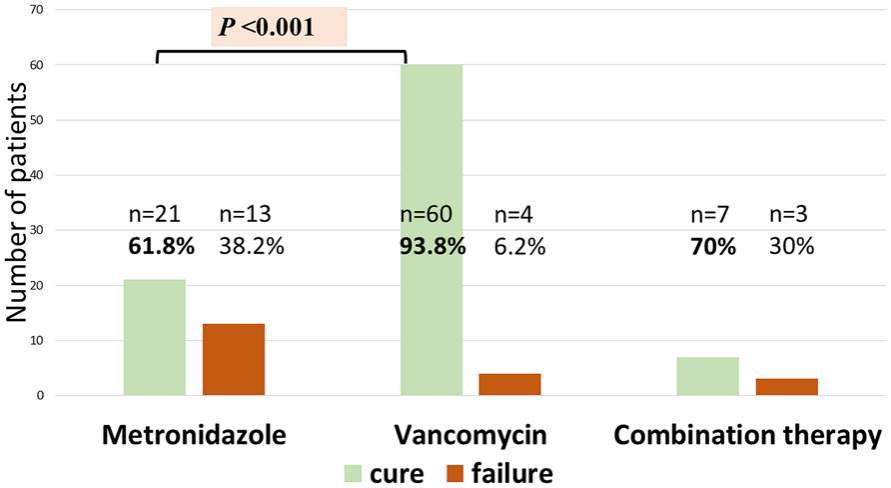
Efficacy of the first-line treatment of *Clostridioides difficile* infection. The columns present absolute numbers of patients who responded (first column—cure) and did not respond (second column—failure) to metronidazole, vancomycin, or combination therapy, respectively.

In the study group, 34 patients (31.2%) were treated with metronidazole as first-line treatment, and 64 patients (58.7%) were treated with vancomycin. One patient (0.9%) was given fidaxomicin, and 10 patients (9.2%) received combination therapy (metronidazole plus vancomycin) due to severe CDI manifestation. Failure of the first-line treatment was more common with metronidazole than with vancomycin (n = 13 [38.2%] and n = 4 [6.2%], respectively; *P* < 0.001). In the combination group, treatment failure was reported for 3 patients (30%) (Fig. 1). Monotherapy with vancomycin was the most common second-line treatment (n = 12; 75%). The remaining options included monotherapy with fidaxomicin (n = 1; 6.3%) and combination therapy with fidaxomicin plus metronidazole (n = 2; 12.6%) or fidaxomicin plus vancomycin (n = 1; 6.3%). In 2 patients, the second-line treatment with vancomycin and fidaxomicin plus metronidazole failed. In the long-term follow-up, at least 14 patients experienced recurrent CDI. Four of these patients were treated successfully with fecal microbiota transplantation.

### Colonization with multidrug-resistant bacteria and use of broad-spectrum antibiotics in patients with *Clostridioides difficile* infection

Colonization with multidrug-resistant bacteria prior to CDI was diagnosed in 71 patients (65.1%). The most common pathogens were vancomycin-resistant Enterococci and bacteria producing extended-spectrum B-lactamases: they were detected in 47 patients (43.1%) and 38 patients (34.9%), respectively. In 17 patients (15.6%), both types of pathogens were detected. Colonization with multidrug-resistant bacteria had no significant impact on the occurrence of GI-GVHD.

In 82 patients (75.2%), broad-spectrum antibiotics were administered directly before the onset of CDI. Of the 26 patients who were exposed only to a single antibacterial agent, 12 (46.1%) received meropenem and 7 (26.9%) received fluoroquinolone (levofloxacin or ciprofloxacin). The remaining 56 patients were exposed to at least 2 antibacterial agents, with many cases of sequential therapies which included meropenem or imipenem in 44 patients (78.6%), cefepime in 14 patients (25%), and piperacillin with tazobactam in 14 patients (25%).

### Associations between *Clostridioides difficile* infection and graft-versus-host disease

Before CDI, aGVHD and cGVHD were diagnosed in 24 and 5 patients, respectively, including 18 patients with gastrointestinal involvement (acute or chronic). We excluded 3 patients with early deaths with CDI from post-CDI GVHD statistical analysis. Exacerbation of GVHD or de novo GVHD after the diagnosis of CDI was reported in 49 patients, including 39 cases of aGVHD (36 patients with grade 2–4) and 10 cases of cGVHD (9 with moderate or severe cGVHD). In 35 of the 49 patients (71%), gastrointestinal involvement was observed. Details concerning number of patients included in analysis and results of statistics are presented in Table 2. We noted statistically significant increase in the incidences of aGVHD and acute GI-GVHD after CDI (*P* = 0.005 and *P* = 0.016, respectively).

**Table 2:** Graft-versus-host disease (GVHD) occurrence before and after *Clostridioides difficile* infection.

	All patients n = 106*	GVHD prior to CDI diagnosis	GVHD after CDI diagnosis and treatment	*P* values
aGVHD	Group n = 94**	24 (25.5%)	39 (41.5%) —Chronic: 5 (5.3%)	P = 0.005
aGVHD grade II-IV		13 (13.8%)	36 (33%)	P < 0.001
acute GI-GVHD		15 (15.9%)	29 (30.9%) —Chronic-GI 2 (2.1%)	P = 0.016
Skin		13		
Liver		5		
cGVHD	Group n = 12***	cGVHD 5 (41.7%) only aGVHD history 4 (33.3%)	5 (41.7%)	P = NS
cGVHD moderate or severe		5 (41.7%)	5 (41.7%)	P = NS
GI-GVHD		3 (25%)	4 (33.3%)	P = NS
Skin		2		
Liver		2		
Other (eyes and mouth)		1		

aGVHD, Acute graft-versus-host disease; cGVHD, Chronic GVHD; GI-GVHD, Gastrointestinal GVHD.

*3Patients with early death not related to CDI or GVHD with active CDI were excluded from post-CDI GVHD assessment.

**Patients with CDI diagnosis peritransplant and in the early posttransplant period without cGVHD diagnosis prior to CDI.

***Patients with CDI diagnosed after Day + 100 (median Day + 231; range 103–740).

Patients receiving combination therapy had a higher rate of GVHD and GI-GVHD than those receiving metronidazole alone (*P* = 0.01 and *P* = 0.007, respectively) and vancomycin alone (*P* = 0.003 and *P* < 0.001, respectively). Moreover, the incidence of GI-GVHD was higher in patients treated with metronidazole as monotherapy or as a component of combination therapy than in the remaining patients (*P* = 0.03). No differences were noted between monotherapy with metronidazole and vancomycin. However, the second-line treatment for CDI, which was administered more frequently in the metronidazole group, was associated with a higher rate of GI-GVHD (*P* < 0.001).

### *Clostridioides difficile* infection: treatment outcomes

**Figure 2:**
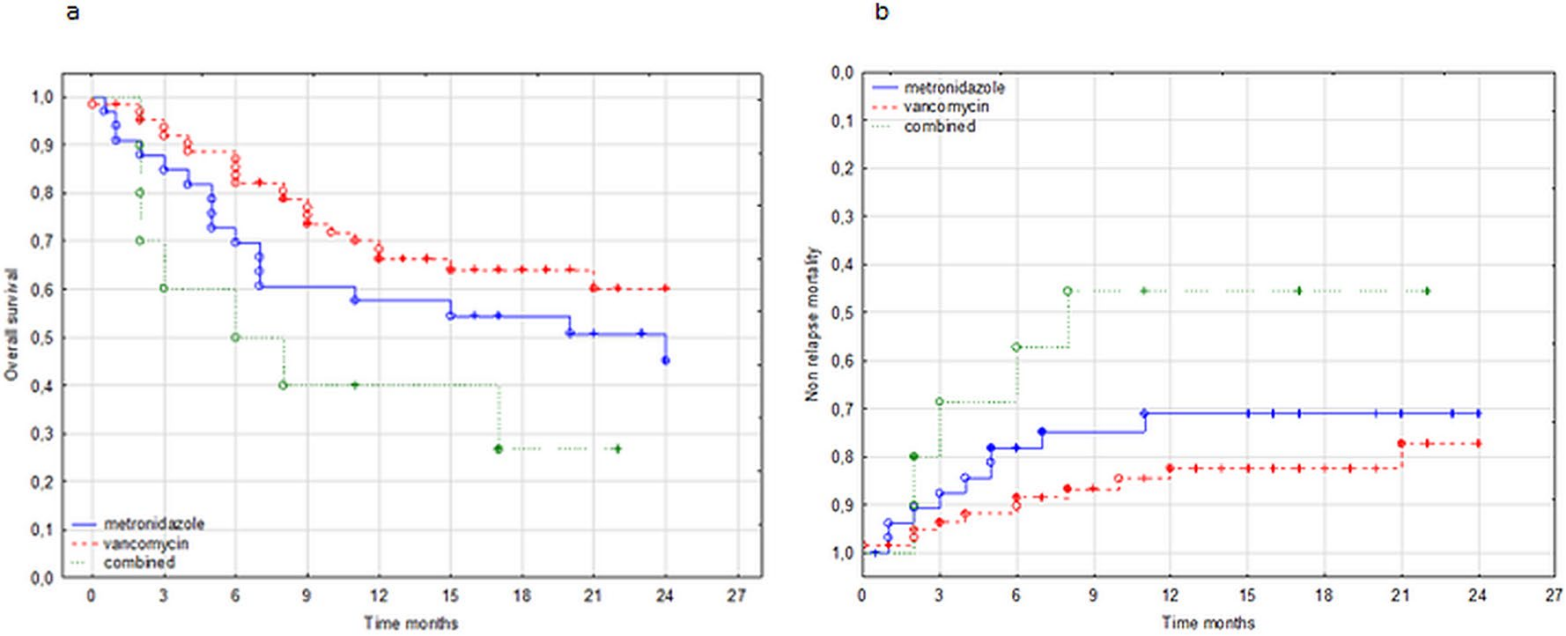
Overall survival (**a**) and non-relapse mortality (**b**) after allogeneic hematopoietic cell transplantation depending on the type of therapy.

The follow-up period was 2 years. Overall survival at 6 months, 1 year, and 2 years after allo-HCT in the study group was 75%, 62%, and 51%, respectively. In patients treated with vancomycin, metronidazole, and combination therapy, OS at 6 months was 81%, 70%, and 56%, respectively and at 1 year—65%, 57%, and 47%, respectively. At 2 years, OS in patients treated with vancomycin and metronidazole was 59% and 45%, respectively. In the group treated with combination therapy, none of the patients survived at 2-year follow-up (Fig. 2a).

**Figure 3:**
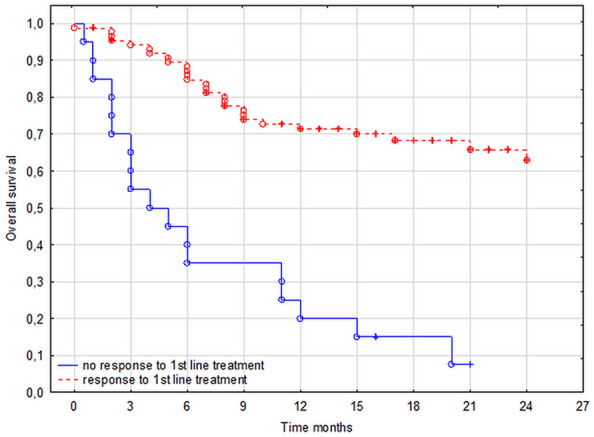
Overall survival depending on the efficacy of the first-line treatment.

Failure of the first-line treatment and a need to switch to the second-line treatment led to lower OS (*P* < 0.05; Fig. 3). The presence of GVHD before CDI (*P* < 0.005) or the development or exacerbation of GVHD after CDI (*P* < 0.05) was associated with increased mortality.

The non-relapse mortality (NRM) rate at 6 months, 1 year, and 2 years was 18%, 25%, and 27%, respectively. The rate differed between patients treated with vancomycin versus combination therapy (*P* < 0.05; Fig. 2b). Five patients died with active CDI within 30 days after allo-HCT, including 2 patients treated with metronidazole, 2 with vancomycin, and 1 with combination therapy. The need to switch to second-line CDI treatment was associated with higher mortality due to GVHD (*P* = 0.001) at 6-month follow-up.

In the multivariate analysis, the occurrence of aGVHD before CDI and the need to switch to the second-line treatment of CDI were predictors of death (HR, 3.19; 95% CI, 1.65–6.16; *P* = 0.009 and HR, 4.83; 95% CI, 2.46–9.47; *P* < 0.001; respectively).

### Clinical practice and treatment outcomes before and after the introduction of noninterventional *Clostridioides difficile* infection protocol

The comparison of patients treated before (retrospective data) and after (prospective data) the introduction of the noninterventional CDI protocol revealed that metronidazole was prescribed in 18 patients (43.9%) versus 16 patients (23.5%), and vancomycin, in 15 patients (22%) versus 49 patients (72%) (*P* = 0.001). The need for second-line treatment was less frequent in patients treated after the introduction of the protocol versus those treated before (*P* < 0.05). However, there were no differences in OS and the rates of GI-GVHD between these groups.

## Discussion

The current study shows that vancomycin is superior to metronidazole as the first-line treatment of CDI in patients after allo-HCT. The administration of metronidazole was associated with a higher rate of treatment failure and worse OS, as compared with vancomycin. Our results align with randomized trials showing that oral metronidazole was associated with poorer outcomes than oral vancomycin^19,26^. Other research groups reported similar data. In a prospective study by Robin et al.^14^, who assessed 23 episodes of CDI in 19 patients with hematological malignancies, metronidazole as the first-line treatment was prescribed in 78% of the patients; vancomycin, in 8%; combination therapy, in 4%; and fidaxomicin, in 8%. The clinical cure was not achieved in 2 patients treated with metronidazole (who subsequently responded to vancomycin), and there were 3 deaths before day 10 of treatment. In a prospective study by Al-Nassir et al.^18^, metronidazole and vancomycin were prescribed for CDI in 65% and 35% of patients, respectively. In the metronidazole group, 29% of patients required a switch to vancomycin due to persistent symptoms. Despite the clear evidence from research, a survey performed by IDWP-EBMT revealed that oral vancomycin or oral metronidazole was used as the first-line treatment for nonsevere CDI in 49.3% and 45.8% of EBMT centers, respectively, and for severe CDI, in 59.7% and 30.6% of the centers, respectively^17^. Our study provides further evidence to encourage transplant centers to follow the latest guidelines of the IDSA/SHEA and ASCTC^15,16^. Low treatment efficacy in patients receiving combination therapy in our study can be explained by a more severe manifestation of CDI in these patients. Parmar et al. reported an even lower cure rate of 38% for the combination therapy consisting of metronidazole and vancomycin in patients with hematological malignancies who underwent allo-HCT^27^.

Our study demonstrated a high NRM rate at 6 months and 1 year, especially in patients with failure of the initial CDI treatment and those with GVHD. However, patients treated with vancomycin showed the highest OS and the lowest NRM as compared with those treated with metronidazole or combination therapy. In a study by Amberge et al., CDI had a negative impact on OS in univariate and multivariate analyses (HR, 1.4; *P* = 0.025 and HR, 1.43; *P* = 0.037; respectively) and on NRM in multivariate analysis (HR, 1.6; *P* = 0.038)^21^. Patients with CDI had a shorter median OS than CDI-negative patients (8 months and 25 months, respectively). In our study group, the risk of death was also significantly associated with the occurrence of aGVHD before CDI. We might hypothesize that CDI-mediated intestinal barrier disruption as well as the severity and duration of inflammation may aggravate the severity of pre-existing GVHD, leading to unfavorable treatment outcomes or to the development of GI-GVHD. Available data on the relationship between CDI and GI-GVHD are conflicting^28,29^. Kamboj et al.^3^ reported no significant associations. On the other hand, Amberge et al. described a higher occurrence of GI-GVHD in patients with symptomatic CDI than in asymptomatic *C. difficile* carriers (HR, 2.5; *P* = 0.02)^21^. Additionally, in a study by Buthani et al., the development of CDI was found to increase the subsequent risk of GI-GVHD (HR 1.92; 95% CI, 1.15–3.19; *P* = 0.01), and this finding was further supported by the multivariate analysis^22^. We agree with the cited authors that acute GVHD and CDI share the same pathogenesis as both pathologies develops in the disturbed microbiota microenvironment and that aGVHD appears closely related to the infection through a bidirectional cause–effect link. Results of our study give additional evidence for significant impact of CDI on exacerbation or developing de novo acute GI-GVHD. The higher incidence of aGVHD and GI-GVHD in patients treated with combination therapy can be attributed to the severity of inflammation and significant dysbiosis associated both with severe CDI and GI-GVHD. In addition, our findings strongly indicates a link between local intestinal processes and systemic inflammation, which promotes immune-mediated GVHD in patients after allo-HCT^30^.

The choice of a therapeutic agent may be crucial for a rapid reduction of inflammation and adverse reactions. Vancomycin achieves high concentrations in the colon due to poor absorption^18,19^. In contrast, metronidazole is absorbed in the intestines, and systemic exposure may lead to neurotoxicity^31^. Additionally, metronidazole can target both the harmful and beneficial strains of *Clostridiales* at the same time, resulting in an imbalance in the gut microbiota. The microbiota and its metabolites can modulate the immune system, intestine epithelial cell integrity, and homeostasis, thus mitigating the severity of GVHD^30,32^. This could explain why exposure to metronidazole may lead to GI-GVHD exacerbation. We did not find significant differences in GI-GVHD rates between patients treated with metronidazole versus vancomycin, probably due to the less frequent use of metronidazole in the prospective stage of the study. However, we indirectly demonstrated this effect by noting a lower remission rate following the first-line treatment with metronidazole, which subsequently necessitated a more frequent use of the second-line agents. The increased need for the second-line treatment had a significant impact on the occurrence of GVHD, particularly in cases with gastrointestinal involvement.

Our study group had a high rate of colonization with multidrug-resistant bacteria, reflecting a well-established link between dysbiosis and a favorable microenvironment for CDI occurrence. In more than 75% of patients with CDI, it was directly preceded by antibiotic therapy for prevention or ongoing infection. This is in line with the study by Robin et al.^14^, who reported that 74% of CDI episodes developed on concomitant non-CDI antibiotics and 78% of patients in whom prophylactic antibiotics were administered. There is also evidence that the exposure to broad-spectrum antibiotics increase the risk of GI-GVHD^33^. These findings question the routine use of antibacterial prophylaxis in patients admitted for allo-HCT, especially those who are colonized with multidrug-resistant bacteria. Although in our current analysis the study protocol made it impossible to assess separately the impact of every broad-spectrum antibiotic on the risk of GI-GVHD, exposure to antibiotics leading to dysbiosis along with CDI should be considered independent risk factors in the GI-GVHD development and have to be treated accordingly in statistical analysis.

The introduction of a noninterventional PALG protocol for CDI treatment was shown to have a beneficial effect on daily clinical practice in transplant centers participating in the study, because vancomycin use as the first-line treatment increased from 22 to 72% of patients. This positive change improved treatment efficacy; however, significant differences in the rates of GI-GVHD were not achieved. Research on a larger population of patients is needed to provide further evidence.

Only a few patients were treated with fidaxomicin, mainly as the second-line treatment. Fidaxomicin is a more potent drug than vancomycin and is associated with a higher cure rate and a lower rate of CDI recurrence in immunocompromised patients^34^. Moreover, fidaxomicin has a less detrimental effect on the intestinal microbiota, and it would be interesting to compare vancomycin and fidaxomicin in the context of GI-GVHD onset and exacerbation^35,36^. In a meta-analysis by Al Momani et al.^37^, fidaxomicin was superior to vancomycin in terms of the recurrence rate but not the cure rate. The authors concluded that fidaxomicin appears to be a significantly better drug, but its cost-effectiveness continues to be controversial.

The limitation of the study includes the retrospective nature of some data collection and the noninterventional study protocol, leading to a nonequal size of the metronidazole and vancomycin groups.

Nevertheless, our study demonstrates that the effectiveness of the first-line CDI treatment significantly affects survival in patients after allo-HCT and plays a crucial role in the development of immune-mediated GI-GVHD. Therefore, once CDI is confirmed, a prompt administration of agents with higher efficacy than metronidazole (vancomycin or fidaxomicin) is recommended, while metronidazole should be used only as an adjunctive IV agent in severe cases of CDI.

## Data Availability

The dataset analyzed during the current study is available from the corresponding author on reasonable request.
